# Bioprocessing of Grape Pomace for Value Added Ingredients with Utilization in Baked Products

**DOI:** 10.3390/foods15010050

**Published:** 2025-12-23

**Authors:** Alexandru Zmuncilă, Carmen Rodica Pop, Anca Corina Fărcaş, Simona Maria Man, Maria Simona Chiș, Alexandra Lițoiu, Adriana Păucean

**Affiliations:** Faculty of Food Science and Technology, University of Agricultural Sciences and Veterinary Medicine, 400372 Cluj-Napoca, Romania; alexandru.zmuncila@student.usamvcluj.ro (A.Z.); carmen-rodica.pop@usamvcluj.ro (C.R.P.); anca.farcas@usamvcluj.ro (A.C.F.); simona.man@usamvcluj.ro (S.M.M.); simona.chis@usamvcluj.ro (M.S.C.); alexandra-andreea.litoiu@student.usamvcluj.ro (A.L.)

**Keywords:** grape pomace, bread fortification, enzyme-assisted extraction, bioprocessing, solid-state fermentation, dough rheology

## Abstract

Bioprocessing grape pomace (GP) presents a sustainable solution aligned with circular economic principles and transforms it into valuable functional ingredients for baked products. This review (2020–2025) synthesizes enzymatic and microbial strategies that modify the fiber–phenolic matrix and improve dough performance. Enzyme-assisted extraction, alone or combined with ultrasound or pressurized liquids, increases extractable polyphenols and antioxidant capacity in GP fractions used as flour substitutions or pre-ferments. Fungal solid-state and lactic fermentations liberate bound phenolic compounds and generate acids and exopolysaccharides. Among these routes, enzyme-assisted extraction and lactic sourdough-type fermentations currently appear the most compatible with bakery-scale implementation, offering substantial phenolic enrichment while relying on relatively simple, food-grade equipment. In current bakery applications, GP is mainly used as crude grape pomace powder, which typically shows higher total phenolics and antioxidant capacity. Moreover, in several models it lowers starch hydrolysis and predicted glycemic index. The practical substitution rate is between 5 and 10% of flour, which balances nutritional gains with processing disadvantages. These can be mitigated by fractionation toward soluble dietary fiber or co-fortification with flours rich in protein and fiber. An additional benefit of these methods includes reduced mycotoxin bioaccessibility in vitro. A key evidence gap is the absence of standardized comparisons between raw and bioprocessed GP in identical formulations. Overall, GP emerges as a promising ingredient for bakery products, while the added technological and nutritional value of bioprocessing remains to be quantified.

## 1. Introduction

**Figure 1:**
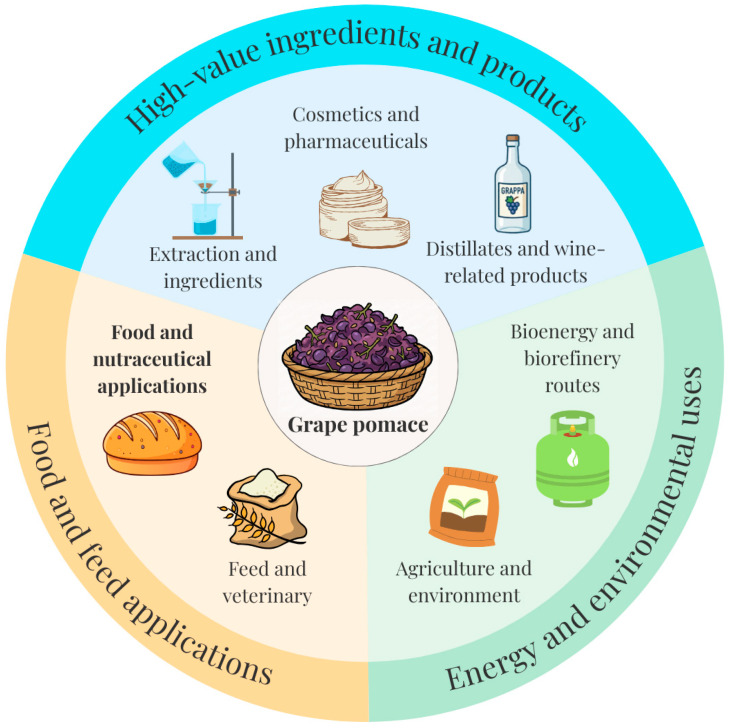
The main valorization routes for grape pomace.

The wine industry generates a considerable quantity of agricultural byproducts. For every 100 L of wine, more than 30% of that volume is created as waste. This corresponds each year to more than 20 million tons of by-products [1]. These include grape pomace, vine leaves, grape stalks, and wine lees. They pose an environmental concern because they are generated in a short amount of time, as wine production is limited in autumn. Moreover, the chemical composition of the winery by-products creates a challenge in discarding them in an environmentally responsible way. Landfilling, although a common practice, has the disadvantage of producing greenhouse gas emissions, as well as surface and groundwater pollution [2]. Another approach includes incineration, which is problematic due to the high moisture content. Instead of disposal, winery by-products can be diverted into multiple valorization routes, including the production of distillates, extraction of tartaric acid and coloring substances, grape seed oil and phenolic extracts, use as animal feed and fermentation substrates, composting and soil amendments, bioenergy production, and the development of high-value food ingredients and nutraceuticals [3]. One example is the supplementation of traditional feed with winery by-products, which has been shown to improve piglet growth and enhance intestinal health [4]. In parallel, grape pomace is increasingly explored as a source of functional ingredients for human foods, particularly baked products, which is the main focus of this review. Figure 1 schematically summarizes the main valorization routes for grape pomace and situates bakery applications within this broader context.

Grape pomace (GP) is a winery byproduct obtained from the pressing of grapes. It contains skins, seeds, pulp residue, and stalk. It is a valuable source of nutrients with a high potential in human nutrition. GP is rich in dietary fibers, proteins and fats, although the exact composition varies depending on the variety of grapes and the terroir [5]. GP is also rich in polyphenols, including anthocyanins, flavonols, and flavan-3-ols. These compounds are often retained within the dietary fiber matrix and are weakly extractable, and this association influences both sensory characteristics and the bioaccessibility of phenolic compounds after baking and digestion [6,7,8]. However, direct substitution of flour with raw grape pomace powder (GPP) often introduces unwanted technological penalties. A high fiber and polyphenol content tends to affect negatively rheological properties of dough [9,10]. It can dilute gluten and introduce particle-induced discontinuities, which often tightens the dough and affects gas stability. This contrast between the nutritional potential of GP and its negative impact on dough structure motivates the search for processing strategies that can unlock GP functionality in bread and other cereal-based bakery products.

Bioprocessing provides an approach for unlocking GP functionality while mitigating processing penalties. Enzyme-assisted extraction (EAE) uses enzymes to break down the cell wall components and release bound compounds, which increases extractable phenolic compounds [11]. Ultrasound-assisted extraction (UAE) and pressurized liquid extraction (PLE) intensify mass transfer via cavitation and solvent penetration, respectively. This is not only efficient but also selective of the targeted compounds [12,13]. Microbial bioprocessing complements these strategies. In solid-state systems, filamentous fungi such as *Aspergillus niger* and *Aspergillus oryzae*, which produce tannase and cellulase, can liberate aglycones and remodel the fiber–phenolic matrix [14]. In submerged or semi-solid fermentations, lactic acid bacteria acidify the matrix. They can also produce exopolysaccharides with water-binding properties that enhance dough viscoelasticity and crumb softness in leavened baked products [15].

Beyond processing performance, bioprocessing has nutritional consequences. It can increase the availability and stability of phenolic compounds, as well as elevating antioxidant capacity at consumption. The interactions between phenolic compounds, starch and protein can reduce starch accessibility, which leads to a lower predicted glycemic index [16]. Additionally, grape pomace has a native microbiota which can initiate spontaneous fermentation. This can lead to an enhanced aroma and increased phenolic accessibility, yet batch variability and safety risks impose a controlled fermentation with defined process parameters [17].

Against this background, a central question and the scope of this review is how specific enzymatic and microbial bioprocessing routes modify GP composition in ways that are relevant for both dough rheology and nutritional outcomes in wheat bread and related bakery systems. In this context, the present work adds to previous reviews on winery by-products and grape pomace by focusing on post-2020 evidence, explicitly linking bioprocesses, such as EAE, UAE, PLE, SSF, SmF to changes in the fiber–phenolic matrix, synthesizing how these changes translate into dough rheology, product quality, glycemic response and food-safety endpoints, and extending the discussion from bread to a broader set of leavened and sweet bakery products.

To construct this review, we performed a structured but narrative literature search in Web of Science, Scopus and Google Scholar for the period 2020–2025, using combinations of the keywords “grape pomace”, “winery by-products”, “bread”, “bakery”, “bioprocessing”, “enzyme-assisted extraction”, “ultrasound-assisted extraction”, “pressurized liquid extraction”, “solid-state fermentation”, “lactic fermentation”, “sourdough” and “dough rheology”. We included peer-reviewed original articles in English that characterized GP composition or polyphenol profiles, applied enzymatic or microbial bioprocessing to GP, and/or incorporated raw or bioprocessed GP (or derived fractions) into cereal-based doughs and bakery products with reported technological and/or nutritional outcomes. Conference abstracts, non-food applications and papers lacking basic methodological detail on the GP matrix or processing conditions were excluded. Pre-2020 studies were cited selectively to provide background on GP composition and health effects when this information was not available in the 2020–2025 body of work.

## 2. Composition and Functional Components

### 2.1. GP Composition

#### 2.1.1. Dietary Fibers

The main fraction of GP is represented by dietary fibers. They make up anywhere from 30% up to 65% of the dry weight. The majority of dietary fibers are insoluble and are formed from structural polysaccharides, such as cellulose and hemicellulose, to which small quantities of pectin and lignin are added [18]. The particularity of this fiber is that it retains a part of the grape’s pomace polyphenols, which results in an antioxidant dietary fiber, capable of delivering natural antioxidants to the intestine.

From a physiological perspective, the beneficial effects of those of normal dietary fiber, but are amplified by polyphenols. Insoluble fibers increase the volume of intestinal transit, maintain regular digestion [19]. Pectin and starch are metabolized in the colon. This generates fatty acids with short chains, such as acetate, propionate, and butyrate. Consequently, this improves colon health, enhances the microbial barrier and improves insulin sensibility. A recent study has linked dietary fibers isolated from a Pinot noir pomace to improved phenolic release and gut-modulating effects, by shifting the microbiota toward a profile favoring *Bifidobacterium* and decreasing the percentage of *Firmicutes* and *Bacteroidetes* by 29–43% [20].

#### 2.1.2. Polyphenols

Polyphenols are an important class of compounds found in GP. They are classified into two major groups: non-flavonoid and flavonoid. The concentration of polyphenols varies depending on the variety of grapes and pedoclimatic conditions, but some studies report anywhere from 6 to 90 mg GAE/g [21,22]. Grape pomace is considered an important source of recoverable polyphenols because over 60% of total phenolic compounds remain in GP after the winemaking process [23].

The most common anthocyanins found in GP are 3-O-glucosides of malvidin, petunidin, cyanidin, peonidin, and delphinidin [24]. The concentration of anthocyanins varies between 84.4 and 131 mg/100 g [25]. GP obtained from red grapes has been found to possess a higher concentration of hydroxycinnamic acid and anthocyanins in comparison to GP obtained from white berries. At the same time, it has much lower levels of catechins, proanthocyanidin dimers, and total flavanols [26]. This is attributed to genetic factors between red and white grapes, as well as the differences in processing both types of grapes in winemaking. Multiple studies have shown that grape anthocyanins possess strong antioxidant and anticancer properties, exhibit neuroprotective effects, help reduce LDL oxidation and can improve insulin sensitivity [27,28,29,30].

Flavonoids represent a major class of polyphenols in grape pomace, encompassing anthocyanins, flavonols, and flavan-3-ols. Recent studies reported total flavonoid contents of 8.4 mg rutin equivalents/g DW in grape pomace extracts, as well values up to 46.2 mg quercetin equivalents/g DW across different cultivars [26,31]. GP also contains a significant quantity of resveratrol, which is a key stilbenic phytoalexin. A recent study has shown that the resveratrol content of 26.5 mg/g in grape pomace extract is comparable to that of other plant sources, such as berries, and nuts [32]. Recent post-2020 studies have linked grape pomace (GP) polyphenols, particularly anthocyanins, flavan-3-ols, flavonols, and stilbenes to clinically and mechanistically relevant outcomes. Across this work, three recurring mechanistic themes emerge: (i) antioxidant and anti-inflammatory activity, including modulation of redox balance and NF-κB signaling; (ii) glycemic regulation and improved insulin sensitivity, sometimes accompanied by changes in bile-acid profiles and gut microbiota; and (iii) vascular and cardiometabolic effects, such as improved endothelial function and atheroprotective biomarker shifts. Table 1 synthesizes representative evidence by compound class, GP fraction, study design, and health effects.

#### 2.1.3. Macronutrients

The proximate composition of grape pomace reported compiled in Table 2 highlights a variability in macronutrient content across grape varieties, processing methods, and sample forms. Moisture values ranged from as low as 3–4 g/100 g in dehydrated pomace [5,40] to more than 20 g/100 g in sun-dried material [41], reflecting differences in drying procedures. Protein content showed a broad distribution between 6 and 13 g/100 g DW, with higher values generally reported for dried whole pomace compared to crude crushed pomace [41,42]. Lipid content demonstrated the widest variability, from 3 to 6 g/100 g in samples with limited seed content to 18–20 g/100 g in pomace powders rich in seeds. Based on technological studies, these higher lipid levels are consistent with seeds representing roughly 38–52% of the dry matter of the solid by-product, corresponding to approximately 3–5% of the original grape mass [5]. Ash levels fluctuated between 1.6 and 9 g/100 g DW, again suggesting the influence of grape cultivar and processing conditions [18,43]. Carbohydrate values vary from 12 to 15 g/100 g DW in dried pomace [5] to as high as 70 g/100 g in fresh crushed pomace [42], where residual sugars and pulp are more abundant.

### 2.2. Natural Microbiota

Fresh grape pomace represents a complex ecological niche that harbors a diverse microbiota inherited primarily from the surface of grape berries and the winemaking environment. This ecosystem is populated by yeasts, lactic acid bacteria, acetic bacteria, and filamentous fungi. Studies have shown that both *Saccharomyces cerevisiae* and a wide range of non-*Saccharomyces* yeasts, including *Hanseniaspora*, *Candida*, and *Metschnikowia* species, remain viable in pomace even after pressing, often at levels sufficient to initiate fermentative activity [45].

The most common LAB species found in grape pomace are *Lactobacillus*, *Pediococcus*, and *Oenococcus*. These bacteria are associated with malolactic fermentation in winemaking. They can persist in pomace due to the presence of residual sugars and moisture [45]. Importantly, recent work indicates that technological steps such as convective or infrared drying at moderate temperatures reduce but do not completely eliminate this microbiota, and viable LAB populations can still be recovered from dried GP-based powders designed as probiotic carriers [46]. LAB metabolism contributes to acidification, as well as the production of organic acids, exopolysaccharides, and bacteriocins. The combination of LAB and yeast activities can introduce a spontaneous fermentation that is similar to traditional natural fermentations such as sourdough or silage. Notably, the native microbiota can initiate the process without external inoculation [45,46,47].

The spontaneous fermentation of GP is of technological significance. Yeast and LAB metabolism can enhance the aromatic complexity of grape pomace, generate organic acids and liberate phenolic compounds through enzymatic hydrolysis. For example, the β-glucosidase activity of certain yeasts facilitates the release of aglycone polyphenols, while LAB may contribute to the breakdown of complex carbohydrates into fermentable sugars, thereby modifying the nutritional profile [47].

Uncontrolled fermentations are inherently inconsistent. Outcomes are influenced by microbial succession, competition, and external parameters such as temperature, pH, and oxygen availability. This unpredictability may lead to inconsistency in metabolite production, microbial safety concerns, or the development of undesirable sensory attributes. These challenges can be mitigated by a careful process monitoring and a strategic combination of natural microbiota with controlled starter cultures to ensure reproducibility and safety [46,47].

## 3. Bioprocessing Techniques

Various bioprocessing methods can be used to improve GP properties. They can break down the fiber matrix, release bound nutrients, and reduce undesirable traits. A key approach is using enzymatic treatments to extract bioactive compounds. Another important method is fungal or bacterial fermentation. Below we summarize recent studies for each method and how they enhance GP for use in cereal doughs and leavened bakery products.

**Figure 2:**
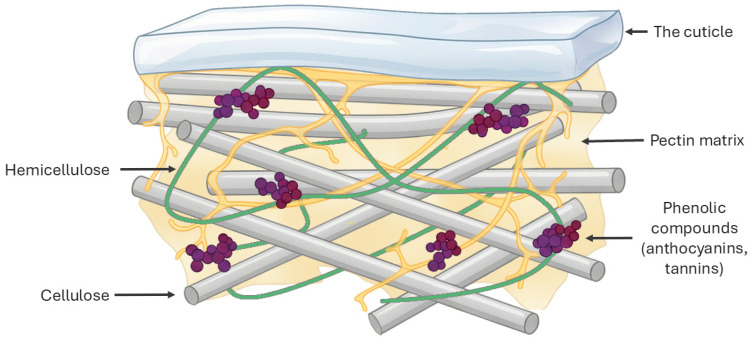
Grape pomace cell wall microstructure and phenolic entrapment.

To contextualize these interventions, Figure 2 illustrates a simplified microstructural model of the grape pomace (GP) cell wall. A pectin matrix embeds cellulose microfibrils that are cross-linked by hemicelluloses covered by a cuticle layer represented as purple clusters associated with the polysaccharide network. These compounds are retained through hydrogen bonding and hydrophobic/π–π interactions, which limits their extractability.

### 3.1. Enzymatic Treatments and Innovative Extractions

**Figure 3:**
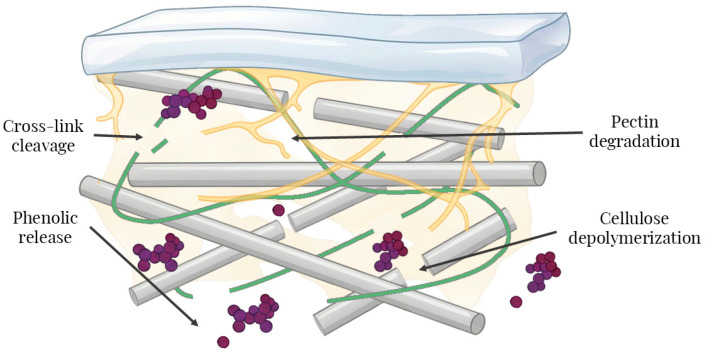
Mechanistic schematic of enzyme-assisted phenolic release from grape pomace.

Enzyme-assisted extraction is a modern technique with great promise. To situate the following results, Figure 3 illustrates enzyme-assisted extraction in grape pomace. Targeted cellulases, pectinases, and hemicellulases weaken the pectin–cellulose–hemicellulose network, open diffusion pathways, and release phenolics previously retained by H-bonding and hydrophobic/π–π interactions. This mechanistic disruption underpins the increases in extractable phenolics and antioxidant capacity.

In parallel with enzyme-assisted extraction (EAE), two other innovative techniques are increasingly applied to GP: ultrasound-assisted extraction (UAE), which relies on acoustic cavitation to disrupt plant tissues and accelerate mass transfer, and pressurized liquid extraction (PLE), which uses elevated temperature and pressure to enhance solvent penetration and shorten extraction times. Although they differ in energy input and selectivity, all three approaches aim to intensify the recovery of polyphenols from complex GP matrices.

EAE is efficient, selective, and eco-friendly. This allows for a targeted extraction of the desired compounds with minimum harm to the environment. Stanek-Wandzel et al. [48] used cellulase, pectinase, and hemicellulose to optimize the extraction conditions of polyphenolic compounds from red whole grape pomace. The cellulase-assisted extract showed the highest total phenolic content (1924 mg GAE/100 g), which is higher than the control of solid–liquid extraction (1717 mg GAE/100 g). Cellulase was also the most effective for catechin extraction, while hemicellulase was the most effective for phenolic acids.

Poblete et al. [49] also evaluated EAE by using tannase and cellulase, and pressurized liquid extraction as a method to optimize polyphenol extraction and antioxidant capacity from pisco grape pomace. PLE showed a higher yield (50.03 mg GAE g^−1^ dw) of total polyphenols than EAE (38.49 mg GAE g^−1^ dw). At the same time, the antioxidant capacity was lower for PLE (342.47 vs. 371.00 μmol TE g^−1^ dw). Machado et al. [50] evaluated EAE with cellulase followed by (PLE) for recovering polyphenolic compounds from *BRS Violeta* grape pomace. The control (PLE alone) extracted the highest TPC (120.1 mg GAE/g), while PLE assisted by EAE was successful in selectively releasing compounds such as gallic acid (1.9-fold), p-coumaric acid, epicatechin, epicatechin gallate, and especially myricetin (10.9-fold).

Ultrasound processing is another emerging method in food processing. It is based on the rupturing of cell walls through high-intensity ultrasonic waves. As such, ultrasound-aided extraction can maximize the yield and minimize the extraction time. Stanek-Wandzel et al. [51] combined ultrasound- and enzyme-assisted extraction to enhance the recovery of polyphenolic compounds from red and white grape pomace. The combined method showed a higher TPC then solid–liquid extraction (2794 vs. 1714 mg GAE/100 g). It also presented the highest antioxidant capacity, preserving anthocyanins such as malvidin-3-glucoside and cyanidin chloride. Balan et al. [52] optimized ultrasound-assisted extraction of polyphenolic compounds from red grape pomace using ethanol and natural deep eutectic solvents (NaDES). The highest total phenolic content was achieved with UAE with ethanol (465.8 mg GAE/100 g dw) compared to NaDES-based UAE (414.0 mg GAE/100 g dw). Ultrasound improved anthocyanin extraction by 1.82 times and doubled the antioxidant capacity. Thus, ultrasound was most effective when combined with ethanol.

Taken together, these studies indicate a functional hierarchy between extraction techniques. Cellulase-based EAE is particularly effective for increasing gallic acid, catechins and some phenolic acids, while leaving a fiber-rich residue suitable for further use [48,50]. UAE, especially in hydroalcoholic systems, maximizes anthocyanin extraction and antioxidant capacity [51,52]. PLE generally provides the highest overall TPC yields, but these extracts do not always show the greatest antioxidant capacity, highlighting a trade-off between yield and functional profile [49,50]. Moreover, most EAE and UAE studies employ whole or skin-rich red GP, whereas seed-enriched matrices are more often targeted by fermentation or seed-focused treatments; this difference in matrix composition partly explains the distinct phenolic profiles obtained.

Across these studies, EAE and UAE typically increase extractable TPC by ≈1.5–2-fold over conventional solid–liquid extraction, whereas SSF of seeds can raise TPC and flavonoids by more than six-fold, albeit at the cost of longer processing times. From an industrial perspective, cellulase-based EAE and hydroalcoholic UAE emerge as the most promising options for bakery value chains: they use food-grade enzymes and relatively simple tanks or ultrasonic units, operate at moderate temperatures, and generate both a phenolic-rich extract and a fiber-rich flour replacer. In contrast, high-pressure PLE requires more specialized equipment and solvent management, making it more suitable for centralized ingredient production than for direct use in bakery plants.

These extraction methods generate GP fractions that can be aligned with bakery formulations. Phenolic-rich extracts obtained by EAE, UAE, or PLE [48,49,50,51,52] can be incorporated into doughs or sourdoughs at low inclusion levels to boost total phenolics and antioxidant capacity. This complements the 5–10% GPP substitutions that increase 5- to 7-fold the TPC in baked bread [53,54], which currently represents the most studied model product. Furthermore, the fiber-rich residue remaining after extraction retain most of the insoluble dietary fiber. It can be milled and used similarly to the isolated SDF/IDF preparations reported by Baskaya-Sezer et al. [55]. This decouples phenolics from bulk fiber and allows a better control of bakery formulations.

### 3.2. Microbial Fermentation of Grape Pomace

Another powerful approach to bioprocess grape pomace is by using microorganisms. Microbial fermentation can modify GP through the action of microbial enzymes and metabolites. The mechanism is based on the enzyme secretion, which breaks down complex carbohydrates, proteins, and polyphenolic complexes. This releases bound phenolic antioxidants, increasing extractable phenolic compounds and antioxidant capacity [56]. Fermentation also creates an acidic environment, which can hydrolyze certain compounds and further release phenolic compounds [57]. Microbial fermentation produces new metabolites, which can enhance the nutritional and sensory profile of GP. For example, bacterial fermentation can produce lactic and acetic acid [58], while yeast fermentation produces ethanol and volatile compounds (e.g., esters, higher alcohols, organic acids) that contribute to the overall aroma [59].

The two main modes for fermenting GP are solid-state fermentation (SSF) and submerged (liquid) fermentation. Below, we explore specific fermentation strategies and their outcomes for grape pomace.

#### 3.2.1. Solid-State Fungal Fermentation

SSF is the cultivation process in which microorganisms are cultivated on moist solid particles. Typically, a bioreactor is filled with the solid substrate and inoculated with a specific microorganism to obtain the desired bioproduct [60]. The most common types of SSF reactors are packed bed reactors, mechanically stirred reactors, tray reactors, and plug flow configurations [61].

Meini et al. [62] applied SSF using *Aspergillus niger* and *Aspergillus oryzae* to optimize the extraction of polyphenolic compounds from Malbec–Tannat whole grape pomace (skins, seeds and pulp residues). The TPC did not significantly increase compared to the non-fermented control. However, the antioxidant capacity was enhanced, reaching 73.7 mmol TE/100 g with *A. niger* and 109.2 mmol TE/100 g with *A. oryzae*. The former produced a balanced enzyme profile consisting of cellulase, tannase and pectinase, while the latter selectively induced tannase, leading to a higher gallic acid release. Both fermented extracts also promoted the growth of *Lactobacillus casei*, confirming their antioxidant and prebiotic potential. Cabezudo et al. [63] also used SFF with *Aspergillus niger* and *Aspergillus oryzae*, with the aim of producing gallic acid and relevant enzymes. The substrate was a combination of whole grape pomace and soybean hull treated with tannic acid. The yield was 0.36 g gallic acid/g tannic acid (7.2 g/L in 72 h), higher than the control without inoculation. *A. oryzae* was also the most effective tannase producer. Both fungi generated extracts with increased antioxidant and tyrosinase-inhibitory activity.

Šelo et al. [64] applied SSF of whole grape pomace using *Rhizopus oryzae* with the aim of enhancing phenolic compound recovery. The content of 11 individual phenolics increased by 1.1–2.5-fold compared to untreated pomace. After 15 days the mass of the pomace decreased by 17.6% with the most notable changes being to the sugars and proteins. Zhao et al. [56] investigated SSF with *Aspergillus niger*, *Monascus anka*, and *Eurotium cristatum* to improve the soluble phenolic profile of grape pomace seeds. SSF increased the TPC by 6.42-fold and the flavonoid content by 6.68-fold compared to the control. This resulted in an increased antioxidant capacity (DPPH = 2.14-fold; ABTS = 3.64-fold).

For bakery applications, and especially in wheat bread doughs, these fractions derived from SSF would be expected to behave differently from raw GPP when included at the 5–10% levels commonly used in wheat breads [53,54]. Partial depolymerization of cell-wall polysaccharides and the formation of phenolic profiles rich in gallic acid after Performing SFF with *Aspergillus* or *Rhizopus* species [62,63,64] leads to a partial depolymerization of cell-wall polysaccharides and the formation of phenolic profiles rich in gallic acid. This should reduce particle hardness and modify water binding, potentially moderating the strong tightening effect and crumb densification observed when untreated GPP is added to dough [53,65]. Additionally, the ability of SSF extracts to promote *Lactobacillus casei* growth [62] suggests that GP treated with SSF could be integrated into sourdough systems both as a fiber carrier and a prebiotic substrate. This can strengthen the LAB activity and supply more extractable phenolics to the final bread. Although such SSF-based ingredients have not yet been systematically analyzed in bakery formulas, they can address the main technological bottlenecks for crude GP.

#### 3.2.2. Submerged/Semi-Solid Fermentation

Submerged fermentation (SmF), also called liquid state-fermentation, cultivates microorganisms in a free-flowing liquid phase inside closed, aerated, and mixed bioreactors. The most common types are stirred-tank, air-lift, or bubble-column units. They allow for a precise control of fermentation parameters, such as aeration/DO, agitation, pH and temperature [66]. Semi-solid fermentation occupies the middle ground between SSF and SmF. The higher water content allows for mixing and mass transfer, but the matrix remains viscous [66].

Torreggiani et al. [47] applied semi-solid sourdough type fermentation using *Lactiplantibacillus plantarum* T0A10 to valorize Primitivo grape pomace (whole wine pomace). The sourdough containing 5% of fermented grape pomace showed increased antioxidant capacity compared to the unfermented control. The DPPH scavenging rose 74% to 95% and ABTS from 0.62 to 1.02 mM Trolox eq. Fermentation also modified the anthocyanin profile, increasing the content of malvidin-3-O-trans-coumaroylglucoside by 17%. At the same time, the fermented extract showed an impressive anti-inflammatory potential. It reduced TNF-α and IL-1β expression by 63% and 60% in Caco2 cells. Chiarini et al. [67] on the other hand applied submerged lactic fermentation to whole Moscato GP to formulate a yogurt-style beverage. After a pH/sugar optimization, GP was inoculated with *Lactiplantibacillus plantarum*. The number of colonies grew from 7 to 9.3 log CFU/mL in 24 h. At the same time, the pH acidified to pH 4.1–4.2 and the lactic acid increased 6-fold. Fermentation also facilitated the increase in esters and alcohols. The viable counts remained up to 8–9 log CFU/mL through 14 days at 5 °C.

Akbulut et al. [68] applied submerged mixed fermentation, by using a yeast pre-fermentation followed by lactic fermentation to produce shalgam juice from blends of black carrot and black grape pomace, where the pomace fraction mainly contributes skins and seeds. At the end of the fermentation, grape pomace increased TPC (up to 1102 mg GAE/L), while raising the content of resveratrol to 198 µg/L on day 9. Antioxidant tests showed that DPPH tended to decrease over time, whereas ABTS increased during the fermentation. Trossolo et al. [69] studied submerged fermentation of a hydrated blend of wine pomace (15% *w*/*w*) and *Chlorella vulgaris* (15% *w*/*w*) for 72 h at 30 °C. The synergic fermentation allowed the LAB to overcome the inhibition usually seen in pomace fermentation. In the low-MW aqueous fraction, ABTS increased from 0.69 to 3.10 mmol Trolox eq g^−1^ with *Weissella cibaria* P9, while DDPH rose from 1.04 to 2.43 mmol BHT 100 g^−1^. This can be attributed to changes in phenolic profile and to the fact that individual phenolics and their synergistic or antagonistic interactions (e.g., catechin vs. chlorogenic acid, or catechin–resveratrol vs. gallic acid–resveratrol) can differentially influence the DPPH and ABTS responses. The fermentation also increased protein quality, with in vitro protein digestibility (IVPD) rising from 93.72% to 96.41–98.03%, and protein digestibility-corrected amino acid score (PDCAAS) from 0.57 to 0.60–0.62. The phenolic profile has also undergone modifications, with a clear shift towards flavanols and flavonols.

From a bakery perspective, these submerged and semi-solid elevate GP to a functional sourdough-type ingredient. Lactic fermentations acidify the matrix, generate lactic and acetic acid, stabilize LAB populations, and increase the proportion of soluble and phenolic compounds [47,67,68,69]. When fermented GP is integrated into wheat doughs it acts as a source of pre-acidified water, phenolic antioxidants, and active LAB. Formulations based on GP pre-ferments can be designed and dosed in analogy with conventional sourdoughs. In practical terms this means taking into consideration the contribution to total hydration and preferment percentage. This offers a route to exploit the improved phenolic bioaccesibility and potential glycemic attenuation associated with fermented GP in bread and other leavened bakery products. Key configurations and outcomes of these bioprocesses are summarized in Table 3.

Across the bioprocessing routes reviewed, some comparative patterns can already be extracted in terms of performance, scalability, and likely cost. Enzyme-assisted extraction with cellulases, pectinases, or hemicellulases [48,49,50] typically provides moderate-to-high increases in total phenolic content at mild temperatures and can be implemented in stirred-tank equipment already familiar to juice and wine processors, but requires food-grade enzymes and relatively long contact times, which confines its use mainly to higher-value extracts. Pressurized liquid extraction [49,50] offers the fastest cycles and the highest phenolic yields per unit time, but demands pressure-rated units and tighter process control, which raises capital and operating costs and makes this option more realistic for centralized extract production than for direct use in bakeries. Ultrasound-assisted extraction with hydroalcoholic systems or natural deep eutectic solvents [52] occupies an intermediate position: it markedly improves anthocyanin and antioxidant extraction and reduces process time, while requiring investment in ultrasonic reactors and management of cavitation-induced heating.

Solid-state fungal fermentations on whole pomace [56,62,64] are technically simple and use low-cost tray or packed-bed reactors, but are slower, more sensitive to contamination, and yield phenolic profiles that depend strongly on strain and substrate; their main advantage lies in producing antioxidant and prebiotic fractions or enzyme-rich extracts that can later be incorporated into cereal matrices. Semi-solid and submerged lactic fermentations [47,67,69] are closest to existing sourdough technology, since they can be run in standard bakery tanks and integrated as GP-based preferments; they add value primarily by improving phenolic bioaccessibility, acidification, and functional metabolite profiles rather than by maximizing extraction yield. Taken together, current data suggest that EAE, UAE, and PLE are best positioned for generating concentrated GP extracts, whereas SSF and lactic fermentations appear more scalable for direct inclusion of bioprocessed GP into dough systems, provided that process control and food-safety requirements are explicitly addressed.

## 4. Application of Grape Pomace in Bakery Products

### 4.1. Formulation Approaches for Grape Pomace in Bread and Other Bakery Products

Formulation routes span direct flour substitution with raw GPP, fractionation of GP into functional fiber components, and co-fortification with complementary flours. In addition, more recent approaches include the incorporation of GP-derived extracts and fermented GP pre-ferments into bakery formulations, which allow a partial decoupling between phenolic loading and fiber/particle effects. Together, these routes cover the main strategies used in recent GP-fortified breads, breadsticks, cakes, muffins, biscuits and cereal bars [53,54,65,70,71,72,73].

**Figure 4:**
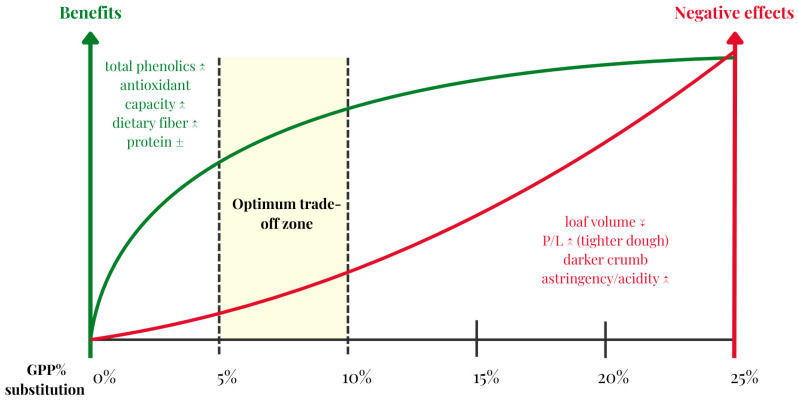
Dose–response of grape pomace powder (GPP) in bread and other bakery products.

Figure 4 summarizes the dose–response for direct GPP substitution in bread and other bakery products. The curves are based on the dose levels and responses reported by Tolve et al. [53] (0, 5 and 10% GP) and Muñoz-Bernal et al. [54] (8% GP), interpolated to visualize the trade-off between fiber enrichment and specific loaf volume. Moderate inclusion (5–10%) consistently increases dietary fiber, total phenolics, and antioxidant capacity, while higher levels penalize structure and intensify astringency/color shift. The 8% level emerges as a practical optimum, delivering nutritional gains with minimal texture or liking trade-offs.

The most predominant method of incorporating GP in bread formulations is by direct flour substation with raw GPP. In a study substituting 0, 5, and 10% flour, GPP increased total dietary fiber from 2.8 to 6.3 g/100 g and antioxidant properties 5-fold. The drawbacks came at the cost of a more tenacious and less extensible dough, with the P/L ratio (balance between dough tenacity—P, and extensibility—L) changing from 0.73 to 11.75. Loaf volume was also inversely correlated with dose. At the same time overall acceptability remained unchanged at 5–10% (6.6 at 5%; 5.6 at 10%; control 6.5) despite a higher acidity and a “wine” aroma note [53]. Another study investigated a larger variety of GP powder substitutions (8, 10, 12, 15, 25%) and identified 8% as the optimum percentage. At this level of substitution, the content of protein was higher by 7.5%, the fiber content by 6.1% and the total phenolics more than doubled (5.1 vs. 2.1 mg GAE/g in control). While the color of the bread darkened, the texture and the consumer acceptance did not differ from the control [54].

Another method of incorporating GP into bread products is through processing it into fractions. Baskaya-Sezer et al. [55] used isolated soluble and insoluble dietary fiber (SDF/IDF) from grapes at 5% substitution. The most promising bread was developed with SDF, due to similarities to the control. As such, the specific volume decreased from 5.63 to 3.95 cm^3^/g and hardness slightly increased from 288 g to 319 g. Microwave treatment improved hydration and modified the microstructure significantly. SDF showed double the water-holding capacity (7.6 g/g), which modified the mixing behavior.

A promising strategy is co-fortification of GP with alternative flours. Rodríguez et al. [70] studied the functional properties of a bread model with 5% GP flour and 20% amaranth flour. This combination presented better results in protecting phenolic compounds during simulated gastrointestinal digestion. This is likely due to the protection offered by the proteins from amaranth flour. In a separate study, combining GP with pecan shell fiber had the effect of raising ash content (2.1 → 2.7–2.9 g/100 g), protein (10.1 → up to 11.8 g/100 g), total dietary fiber (4.7 → 7.4–14.4 g/100 g), and total phenolics (2.1 → 5.3–31.2 mg GAE/g) vs. control. The co-fortified bread also showed a lower predicted glycemic index than control (in vitro GI 0.770–0.81 vs. 1.00) [71].

Beyond yeast-leavened bread, GP has also been integrated into other bakery products. In sponge cakes, Nakov et al. [72] replaced bread wheat flour with 4–10% grape pomace powder and observed gradual increases in ash, lipid, protein, total fiber, free phenolics, anthocyanins and total polyphenols, together with higher DPPH and FRAP antioxidant capacity and lower moisture and pH. The phenolic acids and flavonoids content rose from about 4.1 mg/kg dry matter in the control to 26–60 mg/kg in GPP cakes, while the 4% formulation showed the best sensory quality, indicating that low-to-moderate GPP levels can substantially enhance nutritional value without compromising overall cake acceptability.

Muffins provide another example where higher GPP levels can be tolerated due to the higher sugar and fat content. Troilo et al. [18] formulated muffins in which 15% of wheat flour was replaced with grape pomace flour and showed that all GPP muffins had approximately 3–4 g total dietary fiberr/100 g and 0.6–0.7 mg phenolics/g, while monomeric anthocyanins were around 25–28 µg/g. Finer GPP particles (150–212 µm) increased TPC and ABTS antioxidant capacity (up to ≈2.5 µmol Trolox equivalents/g) but also increased crumb hardness compared with coarser particles, highlighting the role of milling fineness on both functionality and texture. At a broader bakery scale, Antoniolli et al. [73] formulated muffins (5% GP), biscuits (10% GP) and cereal bars (10% GP) with Malbec grape pomace powder and compared them with unsupplemented controls. Across all three products, GP incorporation increased total dietary fiber by approximately six- to seven-fold and led to marked rises in total phenolic content and ORAC antiradical capacity, while concomitantly reducing carbohydrate content. Sensory evaluation showed that GP-enriched muffins, cereal bars and biscuits remained well accepted by consumers, with the main perceived differences being a darker color and a more intense, grape-derived flavor rather than major defects in texture.

Together with the available bread data, these results indicate that direct flour substitution and co-fortification strategies using grape pomace can be extended from bread to a wider range of bakery products, although the maximum feasible GP level in each matrix is ultimately constrained by texture and flavor acceptability.

### 4.2. Compositional, Nutritional, and Sensory Effects

Here we synthesize effects on phenolics, antioxidant capacity, starch digestibility/glycemia, and consumer response.

Across breads, cakes, muffins, biscuits and cereal bars, a consistent pattern emerges: GPP additions raise total dietary fiber, ash, and phenolic content while slightly lowering energy density. In cakes with 4–10% GPP, phenolic acids and flavonoids increased from about 4 mg/kg dry matter in the control to 26–61 mg/kg, along with marked increases in DPPH and FRAP antioxidant responses [72]. In muffins containing 15% GPP, total dietary fibre reached ≈3–4 g/100 g and extractable phenolics ≈0.6–0.7 mg/g, with smaller GPP particles delivering higher ABTS and DPPH values at the expense of somewhat greater hardness [18]. In the muffins, cereal bars and biscuits studied by Antoniolli et al. (2024), GPP enrichment produced about six-fold higher dietary fiber and roughly doubled total phenolics and radical-scavenging capacity compared with the respective controls, while keeping protein and fat in ranges typical for the product categories [73]. Across multiple bread studies, dietary fiber and polyphenols increase substantially with GP. Bread with 5–10% GPP shows a direct correlation between the nutritional benefits and the dose. TPC rose 7.13-fold, antioxidant capacity by 7.88-fold, and dietary fiber by 2.25-fold [53]. The 8% GPP optimum reported in [54] showed gains in protein (+7.5%), fiber (+6.1%), and TPC (from 2.1 to 5.1 mg GAE/g) without significant compromises to texture or overall liking. This supports the feasibility of an enhanced nutritional value with no sensory downsides.

Rocchetti et al. [74] studied the phenolic bioaccessibility and glycemic modulation of bread enriched with GPP. UHPLC-QTOF analysis showed that 5% and 10% GPP breads contained more total phenolics than control (63.8 → 107.0 → 127.8 mg/100 g). Anthocyanin content rose proportionally from 0 mg/100 g to 21.0 and 35.8 mg/100 g, respectively. During in vitro digestion, small intestine anthocyanin bioaccesibility was between 20 and 28%. Starch hydrolysis and predicted glycemic index fell with GPP from 94.2 for the control to 88.9 and 82.7 for the 5% and 10% formulation, respectively. Breads co-fortified with GP and pecan shell also lowered predicted glycemic index to 0.77–0.81 (control 1.00). The intestinal glucose AUC fell by 19–23%. Scanning electron microscopy showed that pores were smaller (0.74 mm vs. 0.54–0.61 mm). The number of micro-pores was significantly higher, with only 60 in control and more than 1230 in formulation with GP and pecan shell. This indicates altered starch–gluten interactions, which aligns with a reduced starch accessibility and lower predicted glycemic index [71]. Likewise, the GP + amaranth bread exhibited improved post simulated gastrointestinal digestion antioxidant capacity relative to single-ingredient breads. This is linked to protective interactions during digestion [70].

Sensory studies converge on the conclusion that moderate GP inclusion is acceptable. At 5–10% GPP, bread shows increased acidity and astringency and with aroma notes typical of grape/wine. Hedonic and texture scores do not decrease significantly, despite a darker crumb. This, however, does not decrease the overall acceptability compared to control [53,54]. For non-bread bakery products, similar patterns are observed: cakes with 4–10% GPP retained acceptable sensory profiles with the 4% level showing the highest overall quality [72], while GPP muffins, biscuits, and cereal bars remained well accepted despite darker color and more pronounced grape-derived notes when fiber and phenolics were markedly increased [73]. A dedicated consumer survey (n = 250) on bread enriched with winery by-products found that acceptance clusters are driven by consumer sensitivity and attitudes, rather than socio-economic factors [75]. This implies that clear communication on health and sustainability can meaningfully skew acceptance towards these products. Table 4 summarizes the proximate composition and key technological outcomes of grape pomace–fortified bakery products reported in recent studies.

### 4.3. Stability, Safety and Processing Performance

Beyond compositional and sensory outcomes, the application of GP at scale requires attention to dough handling, shelf-life, and safety. Here we summarize evidence on processing performance (rheology/microstructure), clean-label shelf-life strategies, and mitigation of mycotoxin bioaccessibility.

Across bread and lean-dough systems (bread, breadsticks), some trends are robust: GPP consistently tightens dough by increasing tenacity and reducing extensibility, primarily through competitive water binding by the fiber-rich particles and physical disruption of the gluten network, darkens crust and crumb, and raises TPC and antioxidant capacity. In contrast, effects on water absorption and proofing time are matrix-dependent and influenced by GPP particle size and dehydration conditions as well as sugar and fat levels, with milling fineness and drying temperature emerging as two major uncontrolled sources of variability in the current literature. This helps to explain why some studies report slightly reduced water absorption at a given GPP level, particularly with very fine powders, whereas others describe a modest increase when coarser, more fibrous GPP is used [18].

Technologically, GP has the behavior of a high-fiber, polyphenol rich particulate that competes for water and interacts with gluten. This reshapes the dough and the loaf characteristics. From a rheological perspective, the addition of 5–10% GPP increases water absorption by up to 5%, although the response depends on the matrix. At the same time, extensibility and deformation energy fell, while proofing and loaf volume declined with dose [53]. The broader dose study confirmed that a higher percentage of GPP substitution increases crumb densification and volume loss. At 8% the volume/texture losses can be minimized with careful hydration and mixing, and overall texture can remain comparable to control. Color change is consistent and expected: crumb and crust darken (brown–purple hues) with dose due to anthocyanins/tannins [54].

Findings in lean dough systems (breadsticks) show similar results to other studies with GPP substitution. At 5–10% GPP, water absorption increased from 58.9% to 60.0%, tenacity P doubled to 215 mm and extensibility fell more than 4 times to 25 mm. Volume and specific volume declined 2.04-fold and 2.09-fold, respectively. Meanwhile, phenolic content increased from 72 to 172 mg GAR/100 g and antioxidant capacity rose more than 5 times (ABTS 234 → 1139 μM TE/100 g) [65]. This data confirms that GP has the effect of tightening dough.

Mechanistically, microstructural imaging shows that GP particles disrupt gluten network continuity. This means that there are thinner strands and more exposed starch directly corelated to the dose. At the same time, stability rose from 6.2 to 9.6 min and development time from 3.2 to 5.2 min for 10–15% GPP, but collapsed at 20% [78]. Breads with 5% isolated SDF is less disruptive and closer to control behavior than IDF. Microwave treatment nearly doubled SDF water-holding capacity, from 3.9 to 7.6 g/g and improved dough stability to 3.9 min [55]. This underlines that particle size, hydration kinetics, and fiber type are essential to optimize processability.

Lou et al. [78] studied rheological and microstructural properties of doughs made with 5–20% GPP from Cabernet Sauvignon. In contrast to previously mentioned studies, here the resulting dough showed a slightly reduced water absorption, lowering it from 67.5% to 65.5%. This can be explained by small particle size of GPP, which increases the overall surface area. Mixing time rose from 6.20 min to 8.5 min, while development time increased from 3.17 min to 4.1 min at 5–15%. Small amplitude rheology showed G′ and G″ lower and tan δ higher versus control. This means that the gluten network is weaker but is still elastic-dominated, which was confirmed by scanning electron microscopy.

Formulation and process aids can be used to stabilize bread systems at scale. In an industrial “pan bauletto” study, the combination of 2% GP with 2% citrus pectin modified water binding from 42.1% to 40.3% and crumb water activity from 0.937 to 0.926. It also increased leavening volume from 230% to 268% and decreased dough density by 0.03 g/cm^3^. Moreover, the decrease in pH from 5.84 to 4.88 and the 2-fold increase in phenolics enabled a better shelf-life, potentially without the use of preservatives [76].

In addition to water activity and pH management in industrial bread, GP may also support safety outcomes during digestion. In bread models spiked with ochratoxin A, the addition of 2% GP lowered intestinal bioaccessibility of the toxin from 94% to 81%. Moreover, the addition showed cytoprotection, with Caco-2 viability rising by 7–27% and mitochondrial mass decreasing by 24% [77]. From a practical standpoint, this reduction in OTA bioaccessibility is particularly relevant for wholegrain and long shelf-life baked products, where mycotoxin contamination remains a recurrent concern. The fact that relatively low GP levels (around 2% of the flour basis) already decreased OTA bioaccessibility and attenuated cytotoxicity in an intestinal cell model suggests that GP could act as a complementary mitigation measure alongside conventional controls such as raw-material selection and process hygiene, adding a concrete safety-related benefit to its nutritional and technological roles.

## 5. Limitations of the Current Evidence

Grape pomace presents a wide range in the contents of moisture, protein, lipids, ash, and dietary fiber due to variability in origin, dehydration strategy, and fractionation yields (Table 2). This heterogeneity propagates into bread and other bakery systems as differences in water absorption, P/L, W (overall dough strength), and microstructure, as discussed in Section 4. Study designs also differ in particle size distributions and hydration/mixing schedules, limiting cross-study comparability of loaf volume and texture outcomes. Although standardized in vitro digestion frameworks exist for bioaccessibility assessment [79], they are not applied consistently in GP-fortified bakery studies, and most data still comes from bread studies.

In Section 3, we discussed that EAE/UAE/PLE and SSF/SmF can increase extractable phenolic compounds and antioxidant capacity. These methods are also effective at selective enrichment with specific compounds like gallic acid and anthocyanins, as well as anti-inflammatory effects. However, application into bread and other bakery formulations is only partially developed. Fermenting GP for sourdough fermentation with LAB inoculation demonstrates antioxidant gains but imparts penalties to the volume and color [47]. On the other hand, thermal processing steps, such as mixing and baking can decrease the anthocyanin content modulated by pH and time-temperature profiles [80]. Nevertheless, there are no trials analyzing the differences between raw and bioprocessed GP within identical dough and bake protocols. In addition, non-bread bakery products (cakes, muffins, biscuits, cereal bars) have so far been investigated in a small number of heterogeneous formulations, often with limited reporting on rheological behavior or in vitro digestion, which restricts the generalizability of current findings.

Native GP microbiota and spontaneous fermentation can enhance aroma and liberate aglycone polyphenols. At the same time, they introduce batch variability and can lead to safety risks without a defined process control. Research on other fruit by-products indicated that spontaneous fermentation can enhance safety and nutritional traits when monitored [81]. Moreover, methods such as hydrostatic-pressure can sanitize and stabilize wine pomace while preserving phenolics [82]. Ensiling with *Lactiplantibacillus plantarum* alters polyphenolic profiles and improves in vitro digestibility [83]. Still, there is no clear pathway defining safety plans and procedures for integrating GP, both raw and processed into bakery products at an industrial scale.

## 6. Conclusions and Future Perspectives

Bioprocessing of grape pomace provides a viable route to transform winery by-products into value-added ingredients for bakery products. This approach leverages its high dietary fiber content and rich polyphenolic profile. Processes such as enzyme-assisted extraction, ultrasound-assisted extraction and pressurized liquid extraction increase the extractable phenolic content and antioxidant capacity. At the same time, solid state fermentation and submerged fermentation modulate phenolic release and produce functional metabolites. For this reason, GP can be considered a functional ingredient. Taken together, current data underline a clear compositional potential: GP systematically enhances fiber and phenolic supply across breads and other bakery matrices, while bioprocessing offers additional control over phenolic profile and bioaccessibility.

Direct flour substitution with GPP at 5–10% has presented an elevated TPC, antioxidant capacity and dietary fiber content. Moreover, sensory analysis has shown no significant difference compared to control. Co-fortification strategies with amaranth flour or pecan shell fiber have protected phenolic compounds during digestion and reduced starch hydrolysis. Moreover, they have shown a capacity of lowering predicted glycemic index by aligning microstructural changes with attenuated glucose release. These results consistently indicate that, within moderate inclusion ranges and with suitable co-ingredients, GP can deliver nutritional enhancement without major penalties in consumer acceptance, especially in breads and sweet bakery products.

When used as an ingredient, grape pomace tends to compete for water and interacts with gluten. Across studies, water absorption increases modestly, with mixing and development time lengthening. Moreover, extensibility and loaf volume have decreased with dose. Small-amplitude oscillatory shear (SAOS) indicates a weaker yet still elastic-dominated network. This can be attributed to a disrupted gluten continuity and increased exposed starch. Another important aspect is fractionation. SDF treated with microwave improved dough hydration and stability. Penalties can also be partially offset by process aids, such as the addition of citrus pectic. This improves leavening, lowers water activity and reduces pH, which suggests a longer shelf-life. In practice, these observations define a second major theme: processing penalties and their mitigation, where particle size, fiber type and process aids determine how far GP levels can be pushed before texture and volume become unacceptable.

Future work should focus on the gap identified in Section 3 by comparing raw versus bioprocessed GP under identical dough and bake protocols. As such, research should focus on retention of anthocyanins, antioxidant capacity and the process penalties to dough rheology and sensory characteristics. Further research should be performed in LAB-inoculated sourdough in order to optimize the functional properties. The role of native microbiota and spontaneous fermentation should be explored with defined safety plans. This should include stabilization steps and ensiling strategies that modulate polyphenols and digestibility.

## Figures and Tables

**Table 1:** Polyphenols identified in grape pomace (post-2020) and reported health effects.

Compound/Class	Main GP Fraction	Reported Health Effects/Outcomes	Model/Design	Source
Proanthocyanidins (PACs)	Seeds >> skins; often bound to insoluble fiber	Improved insulin sensitivity in at-risk adults; suggests metabolic benefits partly independent of major microbiota shifts.	Randomized cross-over clinical trial (n = 49)	[33]
Proanthocyanidins (PACs)	Seeds, skins	Short-term glycemic improvement; bile-acid shift linked to glucose regulation.	Human meta-omics intervention (n = 27)	[34]
Anthocyanins (e.g., malvidin-3-O-glucoside)	Skins (red cultivars)	Anti-inflammatory activity via NF-κB pathway; strong antioxidant capacity (ORAC/FRAP).	In vitro (colon epithelial reporter line)	[35]
Phenolic acids (gallic, caffeic, syringic, chlorogenic, p-coumaric)	Seeds and skins	Reduced TBARS/uric acid or protein carbonyls post-meal; supports anti-oxidative, anti-atherogenic potential.	Human acute crossover (n ≈ 18)	[36]
Stilbenes (resveratrol, piceid) within GP polyphenols	Skins (± seeds)	Atheroprotective biomarker shifts (↓ TMAO, ↓ ox-LDL).	Human clinical study (n = 213)	[37]
Polyphenol-rich white GP extract	White grape skins/seeds	Antihypertensive, antioxidant, anti-inflammatory; endothelial NO pathway.	Animal model (rat)	[38]
GP polyphenol extract (mixed)	Primarily skins/seeds	Anti-obesity; gut microbiota modulation; improved metabolic profile.	Animal model (mouse)	[39]

**Table 2:** Proximate composition of grape pomace from recent research studies.

Grape Variety and Location	Sample Form (Research Design)	Moisture (g/100 g)	Protein (g/100 g DW)	Lipids (g/100 g DW)	Ash (g/100 g DW)	Dietary Fiber (g/100 g DW)	Carbohydrates (g/100 g DW)	References
Isabella grape pomace, La Plata, Argentina	Dried whole pomace	3.7	12.0	9.6	5.5	57.5	14.7	[5]
Cabernet grape pomace, Veneto, Italy	Dried whole pomace	6.9	11.6	4.6	7.0	64.5	12.3	[5]
Băbească Neagră, Romania	Oven-dried pomace powder, particle size <125 µm	11.5	8.4	18.3	1.9	57.0	58.2	[43]
Băbească Neagră, Roma-nia	Oven-dried pomace powder, particle size (125–200 µm)	12.0	8.0	19.5	1.7	52.1	59.2	[43]
Băbească Neagră, Romania	Oven-dried pomace powder, particle size (≥200–<300 µm)	11.6	7.7	20.1	1.6	48.2	60.0	[43]
Zinfandel, Italy	Grape pomace flour used for muffins	–	13.0	8.0	9.0	45.0	–	[18]
Syrah, Argentina	GP powder dried at 55 °C	–	10.04	9.30	–	17.55	50.53	[44]
Syrah, Argentina	GP powder dried at 75 °C	–	10.03	9.57	–	23.11	40.09	[44]
Mixed grape pomace, India	Dried whole pomace	20.4	13.17	12.89	2.68	55.28	15.98	[41]
Vinhão, Portugal	Dehydrated GP	3.43	9.85	3.38	8.20	49.37	35.47	[40]
Red grape pomace for biscuits (Northern Italy)	Crude crushed GP (not fully dried)	14.8	6.0	6.1	3.2	55.0	70.0	[42]

**Table 3:** Representative bioprocessing strategies for grape pomace (2020–2025) and relevance for bakery applications.

Study	GP Matrix/ Fraction	Bioprocess Type	Main Compositional Changes vs. Control	Implications for Bakery Products
Stanek-Wandzel et al. [51]	Red grape pomace (whole)	Enzyme-assisted extraction (EAE)	Cellulase-assisted extract showed the highest TPC (1924 vs. 1717 mg GAE/100 g for solid–liquid extraction); cellulase most effective for catechins; hemicellulase for phenolic acids.	Phenolic-rich extracts for low-level addition to dough/sourdough; enzyme-treated residue can act as a fiber-rich flour replacer.
Poblete et al. [49]	Pisco grape pomace (whole, dried)	EAE vs. pressurized liquid extraction (PLE)	PLE gave higher TPC (50.03 vs. 38.49 mg GAE/g dw) but lower antioxidant capacity (342.47 vs. 371.00 μmol TE/g dw) than EAE.	Illustrates yield vs. antioxidant trade-off; extracts can fortify bread, while exhausted pomace provides dietary fiber.
Machado et al. [50]	BRS Violeta grape pomace	Cellulase-assisted EAE followed by PLE	PLE alone extracted the highest TPC (120.1 mg GAE/g), whereas EAE + PLE selectively enriched gallic acid (1.9-fold), p-coumaric acid, epicatechin, epicatechin gallate and myricetin (10.9-fold).	Enables targeted enrichment in specific phenolics for breads with tailored antioxidant profiles.
Balan et al. [52]	Red grape pomace	Ultrasound-assisted extraction (UAE)	UAE with ethanol gave the highest TPC (465.8 vs. 414.0 mg GAE/100 g dw with NaDES); anthocyanin extraction increased 1.82-fold and antioxidant capacity approximately doubled.	Efficient recovery of anthocyanins and antioxidants for color and antioxidant enhancement in GP-enriched breads/sourdoughs.
Meini et al. [62]	Malbec–Tannat grape pomace	Solid-state fermentation (SSF) with Aspergillus niger and A. oryzae	TPC did not increase significantly vs. control, but antioxidant capacity rose to 73.7 and 109.2 mmol TE/100 g; fermented extracts promoted Lactobacillus casei growth.	SSF extracts show strong antioxidant and prebiotic potential; SSF-treated GP is expected to be less harsh as a high-fiber inclusion in dough.
Šelo et al. [64]	Grape pomace (whole)	SSF with Rhizopus oryzae	Eleven phenolics increased 1.1–2.5-fold; pomace mass decreased by 17.6%, with major changes in sugars and proteins.	Partial depolymerisation of cell-wall components may reduce particle hardness and modify water binding at 5–10% GPP inclusion.
Zhao et al. [56]	Grape pomace seeds	SSF with Aspergillus niger, Monascus anka and Eurotium cristatum	TPC increased 6.42-fold; flavonoids 6.68-fold; antioxidant capacity increased (DPPH 2.14-fold; ABTS 3.64-fold) vs. control.	Highly enriched phenolic fractions can be added at low levels to raise TPC and antioxidant capacity without high fiber load.
Torreggiani et al. [47]	Primitivo grape pomace	Semi-solid lactic fermentation (sourdough-type)	Fermentation with Lactiplantibacillus plantarum T0A10 increased DPPH from 74% to 95% and ABTS from 0.62 to 1.02 mM Trolox eq.; shifted the anthocyanin profile; reduced TNF-α and IL-1β expression in Caco-2 cells.	Shows that GP-based sourdoughs can deliver higher antioxidant capacity and anti-inflammatory potential in wheat bread systems.
Trossolo et al. [69]	Hydrated blend of wine pomace and Chlorella vulgaris (15% + 15% *w*/*w*)	Submerged fermentation (SmF)	ABTS increased from 0.69 to 3.10 mmol Trolox eq/g; DPPH from 1.04 to 2.43 mmol BHT/100 g; IVPD from 93.72% to 96.41–98.03%; PDCAAS from 0.57 to 0.60–0.62; phenolic profile shifted towards flavanols and flavonols.	Synergic fermentation improves protein quality and phenolic profile; conceptually suited as a GP-based fermented ingredient for bread pre-ferments.

Abbreviations: GP—grape pomace; GPP—grape pomace powder; TPC—total phenolic content; dw—dry weight; TE—Trolox equivalents; SSF—solid-state fermentation; SmF—submerged fermentation.

**Table 4:** Applications of grape pomace in bakery products (2020–2025) and the composition outcomes.

Product Type	GP Ingredient and Level (on Flour/Formula Basis)	Key Compositional Outcomes in Final Product	Reference
Bread	Lyophilised red GP powder 2% + citrus pectin 2% (both replacing flour) in industrial pan-bauletto bread	Bread dry matter ~58.4–58.6% (similar across treatments); crumb water activity decreased slightly to 0.916–0.929 (0.937 in control); pH decreased from 5.84 to 4.88; phenolic content increased from 0.67 to 1.10 g/kg DM (≈64% ↑).	[76]
	Whole GP powder, 5% of wheat flour	Total dietary fiber (TDF) 3.9 g/100 g DM (control 2.8; +39%); starch 82.9 g/100 g DM (85.5 in control; ↓); TPC 101.5 mg GAE/100 g DM (29.1 in control; ≈3.5-fold ↑).	[53]
	Whole GP powder, 5% of wheat flour	TPC 107.0 mg/100 g FW (63.8 in control; +68%); anthocyanins 21.0 mg/100 g (0 in control).	[74]
	Lyophilised red GP powder, 8% of wheat flour	Moisture 33.6%; protein 7.5%; fat 1.3%; ash 1.9%; carbohydrates 56.1%; dietary fiber 6.1% (mainly insoluble); pH 4.6; TPC 5.1 mg GAE/g; FRAP 10.0 µmol TE/g.	[54]
	Lyophilised GP powder 8% + pecan shell 5% replacing wheat flour	Moisture 31.0% (33.3% in control); protein 8.3 vs. 7.7 g/100 g; fat 6.4 vs. 4.3 g/100 g; carbohydrates 59.4 vs. 63.7 g/100 g; TDF 14.4 vs. 4.7 g/100 g (≈3-fold ↑, mainly insoluble); TPC 31.2 vs. 2.1 mg GAE/g (≈15-fold ↑).	[71]
	Whole GP powder, 10% of wheat flour	TDF 6.3 g/100 g DM (2.8 in control; +125%); starch 75.3 g/100 g DM (↓ vs. control); TPC 207.1 mg GAE/100 g DM (≈7-fold ↑ vs. 29.1).	[53]
	Whole GP powder, 10% of wheat flour	TPC 127.8 mg/100 g FW (≈2-fold ↑ vs. control 63.8); anthocyanins 35.8 mg/100 g.	[74]
	Lyophilised GP powder 2% replacing wheat flour (with or without ochratoxin A contamination)	Intestinal OTA bioaccessibility decreased from 94% (OTA control) to 81% with GP; GP also reduced mitochondrial stress markers in Caco-2 cells.	[77]
Breadsticks	Red GP powder, 5% of wheat flour mix	Protein 13.11 g/100 g (13.63 in control); lipids 4.86 vs. 4.59 g/100 g; starch 69.67 vs. 70.96 g/100 g; TDF 6.05 vs. 3.47 g/100 g (+74%); ash 2.81 vs. 2.50 g/100 g.	[65]
	Red GP powder, 10% of wheat flour mix	Protein 12.92 g/100 g; lipids 5.30 g/100 g; starch 65.71 g/100 g; TDF 8.55 g/100 g (+146% vs. control); ash 3.08 g/100 g.	[65]
Muffins	Lyophilised red GP powder, 5% of wheat flour	Moisture 20.25 vs. 21.51 g/100 g (control); protein 6.6 vs. 7.33 g/100 g; lipids 21.33 vs. 20.33 g/100 g; fiber 1.90 vs. 0.32 g/100 g (~6× ↑); ash 1.22 vs. 0.68 g/100 g; carbohydrates 47.7 vs. 50.9 g/100 g.	[73]
	Red whole GP powder, 20% of wheat flour; coarse fractions (M425/M300, ≥300 µm)	Moisture ~23–25 g/100 g; protein ~9–10 g/100 g; lipids ~23–26 g/100 g; carbohydrates ~35–40 g/100 g; TDF ~3–4 g/100 g; TPC 0.64–0.69 mg/g; anthocyanins 24.5–28.0 µg/g.	[18]
	Red whole GP powder, 20% of wheat flour; fine fractions (M212/M150, ≤300 µm)	Moisture ~23–25 g/100 g; protein ~9–10 g/100 g; lipids ~23–26 g/100 g; carbohydrates ~35–40 g/100 g; TDF ~3–4 g/100 g; TPC 0.64–0.69 mg/g; anthocyanins 24.5–28.0 µg/g.	[18]
Cupcakes/cakes	Whole GP powder, 10% of wheat flour in cupcakes	TPC 53.73 mg GAE/100 g DM vs. 19.56 in control (≈2.7-fold ↑); anthocyanins 26.4 g/kg DM at 10% GP; strong increase in antioxidant capacity (DPPH, FRAP).	[72]
Biscuits	Lyophilised red GP powder, 10% of wheat flour	Fiber 5.83 vs. 0.90 g/100 g (≈6.5-fold ↑); moisture decreased; ash increased vs. control; other proximate components followed typical biscuit profile.	[73]
Cereal bars	Lyophilised red GP powder, 10% of total bar formula	Fiber 3.31 vs. 0.49 g/100 g (≈6.8-fold ↑); moisture reduced; ash increased vs. control; lipids and carbohydrates similar.	[73]

## Data Availability

No new data were created or analyzed in this study. Data sharing is not applicable to this article.

## References

[1] Ferrer-Gallego R., Silva P. (2022). The Wine Industry By-Products: Applications for Food Industry and Health Benefits. Antioxidants.

[2] Ahmad B., Yadav V., Yadav A., Rahman M.U., Yuan W.Z., Li Z., Wang X. (2020). Integrated Biorefinery Approach to Valorize Winery Waste: A Review from Waste to Energy Perspectives. Sci. Total Environ..

[3] Caponio G.R., Minervini F., Tamma G., Gambacorta G., De Angelis M. (2023). Promising Application of Grape Pomace and Its Agri-Food Valorization: Source of Bioactive Molecules with Beneficial Effects. Sustainability.

[4] Costa M.M., Alfaia C.M., Lopes P.A., Pestana J.M., Prates J.A.M. (2022). Grape By-Products as Feedstuff for Pig and Poultry Production. Animals.

[5] Guardianelli L.M., Salinas M.V., Puppo M.C., Hidalgo A., Pasini G. (2025). Nutritional and Antioxidant Valorization of Grape Pomace from Argentinian Vino De La Costa and Italian Cabernet Wines. Foods.

[6] Wang X., Purcaro G., Fan B., Tong L.-T., Liu L., Sun J., Wang F., Wang L. (2024). Antioxidant Dietary Fibre: A Structure-Function Journey. Trends Food Sci. Technol..

[7] Alves G., Lobo L.A., Domingues R.M.C.P., Monteiro M., Perrone D. (2021). Bioaccessibility and Gut Metabolism of Free and Melanoidin-Bound Phenolic Compounds from Coffee and Bread. Front. Nutr..

[8] Kieserling H., de Bruijn W.J.C., Keppler J., Yang J., Sagu S.T., Güterbock D., Rawel H., Schwarz K., Vincken J.-P., Schieber A. (2024). Protein–Phenolic Interactions and Reactions: Discrepancies, Challenges, and Opportunities. Compr. Rev. Food Sci. Food Saf..

[9] Qin W., Pi J., Zhang G. (2022). The Interaction between Tea Polyphenols and Wheat Gluten in Dough Formation and Bread Making. Food Funct..

[10] Wang Z., Ma S., Li L., Huang J. (2022). Effect of Wheat Bran Dietary Fiber on Structural Properties and Hydrolysis Behavior of Gluten after Synergistic Fermentation of Lactobacillus Plantarum and Saccharomyces Cerevisiae. Front. Nutr..

[11] Amulya P.R., Ul Islam R. (2023). Optimization of Enzyme-Assisted Extraction of Anthocyanins from Eggplant (*Solanum melongena* L.) Peel. Food Chem. X.

[12] Yusoff I.M., Mat Taher Z., Rahmat Z., Chua L.S. (2022). A Review of Ultrasound-Assisted Extraction for Plant Bioactive Compounds: Phenolics, Flavonoids, Thymols, Saponins and Proteins. Food Res. Int..

[13] Višnjevec A.M., Barp L., Lucci P., Moret S. (2024). Pressurized Liquid Extraction for the Determination of Bioactive Compounds in Plants with Emphasis on Phenolics. TrAC Trends Anal. Chem..

[14] Pakaweerachat P., Chysirichote T. (2023). Valorization of Tannin Rich Triphala Waste for Simultaneous Tannase and Gallic Acid Production under Solid State Fermentation by *Aspergillus niger*. Chem. Eng. Commun..

[15] Wu J., Han X., Ye M., Li Y., Wang X., Zhong Q. (2023). Exopolysaccharides Synthesized by Lactic Acid Bacteria: Biosynthesis Pathway, Structure-Function Relationship, Structural Modification and Applicability. Crit. Rev. Food Sci. Nutr..

[16] Graça C., Lima A., Raymundo A., Sousa I. (2021). Sourdough Fermentation as a Tool to Improve the Nutritional and Health-Promoting Properties of Its Derived-Products. Fermentation.

[17] Martău G.A., Teleky B.-E., Ranga F., Pop I.D., Vodnar D.C. (2021). Apple Pomace as a Sustainable Substrate in Sourdough Fermentation. Front. Microbiol..

[18] Troilo M., Difonzo G., Paradiso V.M., Pasqualone A., Caponio F. (2022). Grape Pomace as Innovative Flour for the Formulation of Functional Muffins: How Particle Size Affects the Nutritional, Textural and Sensory Properties. Foods.

[19] Xu J., Li Y., Zhao Y., Wang D., Wang W. (2021). Influence of Antioxidant Dietary Fiber on Dough Properties and Bread Qualities: A Review. J. Funct. Foods.

[20] Li Y., Chen X., Wang G., Xu L., Liu Y., Yuan C., Li J. (2025). The Release Patterns and Potential Prebiotic Characteristics of Soluble and Insoluble Dietary Fiber-Bound Polyphenols from Pinot Noir Grape Pomace In Vitro Digestion and Fermentation. Food Chem. X.

[21] Vorobyova V., Skiba M., Horodniuk O., Khrokalo L., Vasyliev G. (2023). Betaine-Based Deep Eutectic Grape Pomace Extract Mediated Synthesis of Silver Nanoparticles with Antibacterial Activities. BioNanoScience.

[22] da Silva D.J., de Oliveira M.M., Wang S.H., Carastan D.J., Rosa D.S. (2022). Designing Antimicrobial Polypropylene Films with Grape Pomace Extract for Food Packaging. Food Packag. Shelf Life.

[23] Milinčić D.D., Stanisavljević N.S., Kostić A.Ž., Soković Bajić S., Kojić M.O., Gašić U.M., Barać M.B., Stanojević S.P., Lj Tešić Ž., Pešić M.B. (2021). Phenolic Compounds and Biopotential of Grape Pomace Extracts from Prokupac Red Grape Variety. LWT.

[24] Onache P.A., Geana E.-I., Ciucure C.T., Florea A., Sumedrea D.I., Ionete R.E., Tița O. (2022). Bioactive Phytochemical Composition of Grape Pomace Resulted from Different White and Red Grape Cultivars. Separations.

[25] Kannampilly N.J., Devadas C.T. (2019). Kinetic Modelling of Anthocyanin Extraction from Grape (*Vitis vinifera*) Using Response Surface Methodology. Int. J. Innov. Technol. Explor. Eng..

[26] Abreu T., Ferreira R., Castilho P.C., Câmara J.S., Teixeira J., Perestrelo R. (2025). Unlocking the Fatty Acid and Antioxidant Profile of Grape Pomace: A Systematic Assessment Across Varieties and Vintages for Its Sustainable Valorization. Molecules.

[27] Wang Y., Tian X., Cheng T., Liu R., Han F. (2024). Anthocyanins and Proanthocyanidins Synergistically Inhibit the Growth of Gastric Cancer Cells in Vitro: Exploring the Potential Physiological Activity of Grape and Red Wine. Nat. Prod. Res..

[28] Zhu R., Tong X., Du Y., Liu J., Xu X., He Y., Wen L., Wang Z. (2024). Improvement of Chlorpyrifos-Induced Cognitive Impairment by Mountain Grape Anthocyanins Based on PI3K/Akt Signaling Pathway. Pestic. Biochem. Physiol..

[29] Tian Z., Li K., Fan D., Zhao Y., Gao X., Ma X., Xu L., Shi Y., Ya F., Zou J. (2021). Dose-Dependent Effects of Anthocyanin Supplementation on Platelet Function in Subjects with Dyslipidemia: A Randomized Clinical Trial. eBioMedicine.

[30] Yang L., Liu Z., Ling W., Wang L., Wang C., Ma J., Peng X., Chen J. (2020). Effect of Anthocyanins Supplementation on Serum IGFBP-4 Fragments and Glycemic Control in Patients with Fasting Hyperglycemia: A Randomized Controlled Trial. Diabetes Metab. Syndr. Obes..

[31] Selim S., Abdel-Megeid N.S., Alhotan R.A., Ebrahim A., Hussein E. (2023). Grape Pomace: Agrifood By-Product with Potential to Enhance Performance, Yolk Quality, Antioxidant Capacity, and Eggshell Ultrastructure in Laying Hens. Vet. Sci..

[32] Punjab S.Y., Muruga L.J., Jyothi N.T. (2023). Extraction and Evaluation of Resveratrol from Grape Pomace Obtained from By-Product of Wineries: A Sustainable and Cost-Effective Source for Anti-Aging Formulations. LIPS Int. J. Interdiscip. Res..

[33] Ramos-Romero S., Martínez-Maqueda D., Hereu M., Amézqueta S., Torres J.L., Pérez-Jiménez J. (2020). Modifications of Gut Microbiota after Grape Pomace Supplementation in Subjects at Cardiometabolic Risk: A Randomized Cross-Over Controlled Clinical Trial. Foods.

[34] Mezhibovsky E., Wu G., Wu Y., Ning Z., Bacalia K., Sadangi S., Patel R., Poulev A., Duran R.M., Macor M. (2025). Grape Polyphenols Reduce Fasting Glucose and Increase Hyocholic Acid in Healthy Humans: A Meta-Omics Study. npj Sci. Food.

[35] Fariña E., Daghero H., Bollati-Fogolín M., Boido E., Cantero J., Moncada-Basualto M., Olea-Azar C., Polticelli F., Paulino M. (2023). Antioxidant Capacity and NF-kB-Mediated Anti-Inflammatory Activity of Six Red Uruguayan Grape Pomaces. Molecules.

[36] Choleva M., Matalliotaki E., Antoniou S., Asimomyti E., Drouka A., Stefani M., Yannakoulia M., Fragopoulou E. (2023). Postprandial Metabolic and Oxidative Stress Responses to Grape Pomace Extract in Healthy Normal and Overweight/Obese Women: A Randomized, Double-Blind, Placebo-Controlled Crossover Study. Nutrients.

[37] Annunziata G., Ciampaglia R., Maisto M., D’Avino M., Caruso D., Tenore G.C., Novellino E. (2021). Taurisolo®, a Grape Pomace Polyphenol Nutraceutical Reducing the Levels of Serum Biomarkers Associated with Atherosclerosis. Front. Cardiovasc. Med..

[38] Pop R.M., Boarescu P.-M., Bocsan C.I., Gherman M.L., Chedea V.S., Jianu E.-M., Roșian Ș.H., Boarescu I., Ranga F., Muntean M.D. (2025). Beneficial Effects of White Grape Pomace in Experimental Dexamethasone-Induced Hypertension. Diseases.

[39] Han Y., Yang C., Tian X., Shi X., Wang H., Li H. (2025). Grape Pomace Polyphenol Extract Alleviates Obesity in Mice and Improves Gut Microbiota and Short Chain Fatty Acids. Foods.

[40] Machado A.R., Voss G.B., Machado M., Paiva J.A.P., Nunes J., Pintado M. (2024). Chemical Characterization of the Cultivar ‘Vinhão’ (*Vitis vinifera* L.) Grape Pomace towards Its Circular Valorisation and Its Health Benefits. Meas. Food.

[41] Subhashini J., Arulnathan N., Thirumalaisamy G., Jagatheesan P.N. (2025). Assessing the Nutritional Content of Grape Pomace and Its Potential Utilization as a Fruit Processing Industry Waste: A Substitute for Livestock Feed. Asian J. Dairy Food Res..

[42] Giosuè A., Siano F., Di Stasio L., Picariello G., Medoro C., Cianciabella M., Giacco R., Predieri S., Vasca E., Vaccaro O. (2024). Turning Wastes into Resources: Red Grape Pomace-Enriched Biscuits with Potential Health-Promoting Properties. Foods.

[43] Spinei M., Oroian M. (2024). Characterization of Băbească Neagră Grape Pomace and Incorporation into Jelly Candy: Evaluation of Phytochemical, Sensory, and Textural Properties. Foods.

[44] Baldán Y., Riveros M., Fabani M.P., Rodriguez R. (2023). Grape Pomace Powder Valorization: A Novel Ingredient to Improve the Nutritional Quality of Gluten-Free Muffins. Biomass Conv. Bioref..

[45] Franco W., Benavides S., Valencia P., Ramírez C., Urtubia A. (2021). Native Yeasts and Lactic Acid Bacteria Isolated from Spontaneous Fermentation of Seven Grape Cultivars from the Maule Region (Chile). Foods.

[46] Anghel L., Milea A.Ș., Constantin O.E., Barbu V., Chițescu C., Enachi E., Râpeanu G., Mocanu G.-D., Stănciuc N. (2023). Dried Grape Pomace with Lactic Acid Bacteria as a Potential Source for Probiotic and Antidiabetic Value-Added Powders. Food Chem. X.

[47] Torreggiani A., Demarinis C., Pinto D., Papale A., Difonzo G., Caponio F., Pontonio E., Verni M., Rizzello C.G. (2023). Up-Cycling Grape Pomace through Sourdough Fermentation: Characterization of Phenolic Compounds, Antioxidant Activity, and Anti-Inflammatory Potential. Antioxidants.

[48] Stanek-Wandzel N., Krzyszowska A., Zarębska M., Gębura K., Wasilewski T., Hordyjewicz-Baran Z., Tomaka M. (2024). Evaluation of Cellulase, Pectinase, and Hemicellulase Effectiveness in Extraction of Phenolic Compounds from Grape Pomace. Int. J. Mol. Sci..

[49] Poblete J., Aranda M., Quispe-Fuentes I. (2025). Efficient Conditions of Enzyme-Assisted Extractions and Pressurized Liquids for Recovering Polyphenols with Antioxidant Capacity from Pisco Grape Pomace as a Sustainable Strategy. Molecules.

[50] Machado T.O.X., de AC Kodel H., Alves dos Santos F., dos Santos Lima M., Costa A.S.G., Oliveira M.B.P.P., Dariva C., dos Santos Estevam C., Fathi F., Souto E.B. (2025). Cellulase-Assisted Extraction Followed by Pressurized Liquid Extraction for Enhanced Recovery of Phenolic Compounds from ‘BRS Violeta’ Grape Pomace. Sep. Purif. Technol..

[51] Stanek-Wandzel N., Zarębska M., Wasilewski T., Hordyjewicz-Baran Z., Krzyszowska A., Gębura K., Tomaka M. (2025). Enhancing Phenolic Compound Recovery from Grape Pomace Residue: Synergistic Approach of Ultrasound- and Enzyme-Assisted Extraction. ACS Omega.

[52] Balan N., Măntăilă S., Râpeanu G., Stănciuc N. (2025). Enhanced Extraction of Bioactive Compounds from Red Grape Pomace: Optimizing Ultrasound-Assisted Extraction with Ethanol and NaDES as Solvents. Antioxidants.

[53] Tolve R., Simonato B., Rainero G., Bianchi F., Rizzi C., Cervini M., Giuberti G. (2021). Wheat Bread Fortification by Grape Pomace Powder: Nutritional, Technological, Antioxidant, and Sensory Properties. Foods.

[54] Muñoz-Bernal Ó.A., Coria-Oliveros A.J., Vazquez-Flores A.A., Subiria-Cueto C.R., De La Rosa L.A., de la Luz Reyes-Vega M., Rodrigo-García J., del Rocio Martinez-Ruiz N., Alvarez-Parrilla E. (2024). Functional and Sensory Evaluation of Bread Made from Wheat Flour Fortified with Wine Byproducts. Food Prod. Process. Nutr..

[55] Baskaya-Sezer D. (2023). The Characteristics of Microwave-Treated Insoluble and Soluble Dietary Fibers from Grape and Their Effects on Bread Quality. Food Sci. Nutr..

[56] Zhao Y., Liu D., Zhang J., Shen J., Cao J., Gu H., Cui M., He L., Chen G., Liu S. (2024). Improving Soluble Phenolic Profile and Antioxidant Activity of Grape Pomace Seeds through Fungal Solid-State Fermentation. Foods.

[57] Xiao X., Li J., Xiong H., Tui W., Zhu Y., Zhang J. (2022). Effect of Extrusion or Fermentation on Physicochemical and Digestive Properties of Barley Powder. Front. Nutr..

[58] Shen Y., Kang B., Lu Y., Du X., Qin C., Li J., Zhao Z., Yu R., Shi S., Han L. (2023). Production of Optical Pure L-Lactic Acid from Cabernet Sauvignon Grape Pomace by Engineered Lactiplantibacillus Plantarum. Front. Energy Res..

[59] Barakat N., Bouajila J., Beaufort S., Rizk Z., Taillandier P., El Rayess Y. (2024). Development of a New Kombucha from Grape Pomace: The Impact of Fermentation Conditions on Composition and Biological Activities. Beverages.

[60] Mitchell D.A., De Lima Luz L.F., Krieger N., Berovič M. (2011). Bioreactors for Solid-State Fermentation. Comprehensive Biotechnology.

[61] Oiza N., Moral-Vico J., Sánchez A., Oviedo E.R., Gea T. (2022). Solid-State Fermentation from Organic Wastes: A New Generation of Bioproducts. Processes.

[62] Meini M.-R., Cabezudo I., Galetto C.S., Romanini D. (2021). Production of Grape Pomace Extracts with Enhanced Antioxidant and Prebiotic Activities through Solid-State Fermentation by *Aspergillus niger* and *Aspergillus oryzae*. Food Biosci..

[63] Cabezudo I., Galetto C.S., Romanini D., Furlán R.L.E., Meini M.R. (2023). Production of Gallic Acid and Relevant Enzymes by Aspergillus Niger and Aspergillus Oryzae in Solid-State Fermentation of Soybean Hull and Grape Pomace. Biomass Conv. Bioref..

[64] Šelo G., Planinić M., Tišma M., Martinović J., Perković G., Bucić-Kojić A. (2023). Bioconversion of Grape Pomace with Rhizopus Oryzae under Solid-State Conditions: Changes in the Chemical Composition and Profile of Phenolic Compounds. Microorganisms.

[65] Rainero G., Bianchi F., Rizzi C., Cervini M., Giuberti G., Simonato B. (2022). Breadstick Fortification with Red Grape Pomace: Effect on Nutritional, Technological and Sensory Properties. J. Sci. Food Agric..

[66] Palladino F., Marcelino P.R.F., Schlogl A.E., José Á.H.M., Rodrigues R.d.C.L.B., Fabrino D.L., Santos I.J.B., Rosa C.A. (2024). Bioreactors: Applications and Innovations for a Sustainable and Healthy Future—A Critical Review. Appl. Sci..

[67] Chiarini E., Alessandria V., Buzzanca D., Giordano M., Seif Zadeh N., Mancuso F., Zeppa G. (2024). Valorization of Fruit By-Products Through Lactic Acid Fermentation for Innovative Beverage Formulation: Microbiological and Physiochemical Effects. Foods.

[68] Akbulut M., Çoklar H., Bulut A.N., Hosseini S.R. (2024). Evaluation of Black Grape Pomace, a Fruit Juice by-Product, in Shalgam Juice Production: Effect on Phenolic Compounds, Anthocyanins, Resveratrol, Tannin, and in Vitro Antioxidant Activity. Food Sci. Nutr..

[69] Trossolo E., Alabiden Tlais A.Z., Tonini S., Filannino P., Gobbetti M., Cagno R.D. (2025). Fermentation of a Wine Pomace and Microalgae Blend to Synergistically Enhance the Functional Value of Protein- and Polyphenol-Rich Matrices. Food Res. Int..

[70] Rodríguez M., Bianchi F., Simonato B., Rizzi C., Fontana A., Tironi V.A. (2024). Exploration of Grape Pomace Peels and Amaranth Flours as Functional Ingredients in the Elaboration of Breads: Phenolic Composition, Bioaccessibility, and Antioxidant Activity. Food Funct..

[71] Subiria-Cueto R., Reyes-Blas H., Olivas-Armendáriz I., Wall-Medrano A., González-Aguilar G.A., de la Rosa L.A., Martínez-Ruiz N.d.R., Alvarez-Parrilla E. (2025). Grape Pomace and Pecan Shell Fortified Bread: The Effect of Dietary Fiber-Phenolic Compounds Interaction on the In Vitro Accessibility of Phenolic Compounds and In Vitro Glycemic Index. Food Chem..

[72] Nakov G., Brandolini A., Hidalgo A., Ivanova N., Stamatovska V., Dimov I. (2020). Effect of Grape Pomace Powder Addition on Chemical, Nutritional and Technological Properties of Cakes. LWT.

[73] Antoniolli A., Becerra L., Piccoli P., Fontana A., Antoniolli A., Becerra L., Piccoli P., Fontana A. (2024). Phenolic, Nutritional and Sensory Characteristics of Bakery Foods Formulated with Grape Pomace. Plants.

[74] Rocchetti G., Rizzi C., Cervini M., Rainero G., Bianchi F., Giuberti G., Lucini L., Simonato B. (2021). Impact of Grape Pomace Powder on the Phenolic Bioaccessibility and on In Vitro Starch Digestibility of Wheat Based Bread. Foods.

[75] Miolla R., Ottomano Palmisano G., Roma R., Caponio F., Difonzo G., De Boni A. (2023). Functional Foods Acceptability: A Consumers’ Survey on Bread Enriched with Oenological By-Products. Foods.

[76] Scappaticci G., Mercanti N., Pieracci Y., Ferrari C., Mangia R., Marianelli A., Macaluso M., Zinnai A. (2024). Bread Improvement with Nutraceutical Ingredients Obtained from Food By-Products: Effect on Quality and Technological Aspects. Foods.

[77] Mangiapelo L., Frangiamone M., Vila-Donat P., Paşca D., Ianni F., Cossignani L., Manyes L. (2024). Grape Pomace as a Novel Functional Ingredient: Mitigating Ochratoxin A Bioaccessibility and Unraveling Cytoprotective Mechanisms In Vitro. Curr. Res. Food Sci..

[78] Lou W., Li B., Nataliya G. (2021). The Influence of Cabernet Sauvignon Wine Grape Pomace Powder Addition on the Rheological and Microstructural Properties of Wheat *x*Dough. CyTA—J. Food.

[79] Wu P., Chen X.D. (2021). Validation of in Vitro Bioaccessibility Assays—A Key Aspect in the Rational Design of Functional Foods towards Tailored Bioavailability. Curr. Opin. Food Sci..

[80] Francavilla A., Joye I.J. (2022). Anthocyanin Content of Crackers and Bread Made with Purple and Blue Wheat Varieties. Molecules.

[81] de Oliveira S.D., de Souza E.L., Araújo C.M., Martins A.C.S., Borges G.d.S.C., Lima M.d.S., Viera V.B., Garcia E.F., da Conceição M.L., de Souza A.L. (2023). Spontaneous Fermentation Improves the Physicochemical Characteristics, Bioactive Compounds, and Antioxidant Activity of Acerola (*Malpighia emarginata* D.C.) and Guava (*Psidium guajava* L.) Fruit Processing by-Products. 3 Biotech.

[82] Ramírez R., Delgado J., Rocha-Pimienta J., Valdés M.E., Martín-Mateos M.J., Ayuso-Yuste M.C. (2023). Preservation of White Wine Pomace by High Hydrostatic Pressure. Heliyon.

[83] De Bellis P., Maggiolino A., Albano C., De Palo P., Blando F. (2022). Ensiling Grape Pomace with and Without Addition of a *Lactiplantibacillus plantarum* Strain: Effect on Polyphenols and Microbiological Characteristics, In Vitro Nutrient Apparent Digestibility, and Gas Emission. Front. Vet. Sci..

